# How Stable, Really? Traditional and Nonlinear Dynamics Approaches to Studying Temporal Fluctuations in Personality and Affect

**DOI:** 10.3390/ijerph19138008

**Published:** 2022-06-29

**Authors:** Alessio Gori, Daniel Dewey, Eleonora Topino, Marco Giannini, David Schuldberg

**Affiliations:** 1 Department of Health Sciences, University of Florence, Via di San Salvi 12, Pad. 26, 50135 Firenze, Italy; giannini@psico.unifi.it; 2 Integrated Psychodynamic Psychotherapy Institute (IPPI), Via Ricasoli 32, 50122 Firenze, Italy; 3 Department of Psychiatry and Behavioral Sciences, Medical University of South Carolina, 171 Ashley Ave Suite 419, # 403, Charleston, SC 29425, USA; daniel.dewey@umconnect.umt.edu; 4 Department of Human Sciences, LUMSA University of Rome, Via della Traspontina 21, 00193 Rome, Italy; eleonora.topino@gmail.com; 5 Department of Psychology, The University of Montana, 32 Campus Dr, Missoula, MT 59812, USA; david.schuldberg@umontana.edu

**Keywords:** personality traits, five-factor model, positive and negative affects, ecological momentary assessment

## Abstract

A pair of quantitative case studies is presented to demonstrate how different approaches to quantifying temporal variability in ratings of traits and affect can provide rich information for personality researchers. Data are presented and analyzed from two college students who completed an Ecological Momentary Assessment protocol sampling ratings of affect and traits up to 24 times daily for one week. Both classical and nonlinear data analytic techniques were applied to the data to summarize and examine the temporal dynamics of both traits and affect. For the purposes of exposition, one Big Five trait rating, extraversion, and the PANAS positive and negative affects, are discussed. The results support previous research demonstrating a high degree of variability in ratings of both traits and affect over time. Analyses using nonlinear and complexity expand on these findings and suggest temporal patterning as well as disorder; implications of phase portraits for understanding variability are discussed. The findings are discussed in light of a processing dynamics approach to resolving the role of variability in understanding personality.

## 1. Introduction

The study of personality traits and individual differences has captured the attention of personality researchers for many years [1,2,3,4,5]. A fundamental assumption underlying a trait perspective on personality has been that humans behave in a relatively consistent manner over time, and that the presence of specific traits can be determined through patterns of those consistent behaviors [6]; the five-factor model provides a widely used contemporary framework for this perspective [7]. However, despite widespread acceptance of the notion of consistency of traits, researchers have continuously struggled with the concept of traits themselves based on persistent evidence that behavior is not always consistent [8,9].

Research has indicated two clear findings regarding the stability and variability of traits: traits have been found to be moderately consistent over long periods of time, and trait consistency increases as participants grow older [10]. This relative consistency co-exists with a great deal of variability in the short term across different situations [8,11] or over time [12,13,14]. Mischel and Shoda [15] attempt to account for variability in personality by contrasting “behavioral dispositions” and “processing dynamics” approaches. The behavioral dispositions approach, termed “’dispositional’ or ‘trait theory’ … posits broad stable traits, factors, or behavioral dispositions as its basic units… stable dispositions that remain invariant across situations… determining a wide range of important behaviors” (p. 231). In contrast, the processing dynamics perspective emphasizes “how the person functions psychologically in terms of the mediating processes that underlie stable individual differences … that can make sense of intra-individual variability across situations” and “the interaction of the specific situation with the social–cognitive–emotional processing system of the individual” (pp. 230–231).

Fleeson and colleagues [12,16,17,18,19,20] argued for a broad resolution of the trait-situation debate that includes a wider notion of what constitutes consistency, acknowledges within-person variability in traits and related behaviors (including over time periods of less than one week), and accepts that variability itself provides useful information. Personality involves both time, as well as, paraphrasing Sullivan [21] (pp. 103–104), enduring patterning.

Variability in emotions has been treated somewhat differently. In contrast to the popularity of dispositional approaches in personality, instability and temporal change have generally been taken as defining components of mood or affect. Larson and Csikszentmihalyi [22] referred to this (in the context of adolescence) as “moodiness.” Affect has been studied using real-time and other self-report techniques that highlight intra-individual variability (e.g., [23,24,25,26,27]); more recently, interest has developed in affect variation in mood disorders (e.g., [28,29,30,31,32,33,34,35]), delinquency symptoms [36], and more broadly, in mental health problems [37]. Because of this tradition, affect can serve as a benchmark for considering the degree of variability in traits. 

### 1.1. Ecological Momentary Assessment (EMA) and the Experience Sampling Method (ESM)

A data collection methodology that enables researchers to examine temporal psychological dynamics empirically in the real world is the experience sampling method (ESM) [38] or ecological momentary assessment (EMA) [39,40,41]. These two terms refer to roughly the same techniques, with ESM applied first to earlier methodologies with diary techniques, generally with prompts from a timer carried by the participant; EMA, a newer term, tends to use automated recording techniques instead of paper and pencil. Stone and Shiffman [29] described that both ESM and EMA are characterized by a data collection that takes place outside the researchers’ laboratory (i.e., it happens in the real world of the participants), where multiple evaluations were repeated according to different factors (e.g., events, time, randomly, etc.) to capture states or behaviors in a way that is as simultaneous as possible with their occurrence [39]. 

The primary advantage of both EMA and ESM is that they offer ways to reduce the effect of recall bias and offer up-to-date information on participants’ cognitive, behavioral, and emotional experiences in their natural environments [42]. Therefore, this allows one to reduce the risk of some biases that are typical of many traditional assessment techniques. Finally, the ESM/EMA methods also allow for the detection of some intervening environmental factors (such as place, time, and interpersonal dynamics), which may be momentary and may be part of an ecological consideration in the analysis of the examined outcomes (e.g., behaviors, symptoms, etc.) [43].

### 1.2. Studying Variability in Traits

Fleeson and Gallagher [12] used ESM to study variations in behaviors related to specific personality traits. Across a number of studies, the researchers measured trait levels by means of traditional self-report, as well as assessing the temporal variability in repeated measurements of behavioral manifestations in terms of traits, assessed by adjectives referring to behavior during the previous half hour. Measures of central tendency (mean, median, mode, a corrected mean) were used as measures of central or overall tendency of these behavior ratings, related to the notion of an act–frequency approach to traits [44]; they use the standard deviation of behaviors across time as a measure of variability [12]. Not surprisingly, they found an individual’s level of a trait (“standing”) generally predicts overall levels of the corresponding behavioral manifestations. Random point measurements of the behavior were also well predicted by trait standing. 

Individuals were also generally highly variable, as indexed by the standard deviation, particularly on behaviors related to extraversion and conscientiousness; within-subject variation was greater than the variation between participants; “the typical individual routinely and regularly expressed most levels of most states” [12] (p. 1104). The standard deviation for each trait also served as an individual difference variable; it was negatively correlated with the levels of the trait itself for emotional stability and positively correlated with behaviors related to intellect. This variability has been a subject of continued interest and has found further confirmation and enrichment in recent studies, which have, for example, considered the associations between trait dynamics and daily hassles [45], as well as the person—environment transactions [46]. Therefore, the field related to the study of personality fluctuations appears extremely fertile, also in the light of the different fields of practical application (e.g., [46]).

### 1.3. Studying Variability in Affect

EMA and ESM have also been used in studying variations in affect, both non-clinical and pathological (e.g., [47,48]). As an example of the former, Larson, Csikszentmihalyi, and Graef [49] used an older Experience Sampling methodology to study variability in adolescents’ mood and examined their results by visual inspection, by “degree of variation” (indexed by standard deviation), “changeability” tapped by low autocorrelations, and also “situational independence,” indexed by a lack of correlation with the person’s contemporaneous activities, also assessed by the ESM method. The indices of variability all behaved somewhat differently. Adolescents were more variable and more changeable than adults in affects, and recovered from extreme moods more quickly, with their moods about equally related to situations. Interestingly, variability, while correlated with stress, intellectual disengagement, and social alienation, was also positively correlated with a measure of creativity. In more recent research, the use of the EMA has also found increasing confirmation in the study of psychopathology, offering important insights for treatment, allowing us to observe the variations in affect associated with problematic behavioral manifestations, such as non-suicidal self-injury (NSSI) [50], loss-of-control eating [51], and substance use [52], to name a few.

### 1.4. Indices of Variability

Across data-collection methodologies and time periods, the mathematical indices of variability studied as parameters for understanding psychological processes and their individual differences have differed extensively in studies of many different psychological characteristics [53,54,55], including both affect and personality-related variables. Many rely on the moment-based standard deviation [24]. Time series methods supply indices of autocorrelations, cross correlations, and trends, as well as analyses of frequency and spectra. Beyond these methods, there are also methods for characterizing the dynamics of trajectories or time series in terms of their patterning and irregularity [53]. Recent research on mood disorders has been interested in approaches to dynamics of affect disorders that utilize concepts from nonlinear dynamics and chaos theory [28,29,30,31,32,33,34,56].

### 1.5. The Present Study

In previous work, we asserted that nonlinear methods can be very useful in the assessment of personality and its temporal dynamics because they allow for the study of patterns that are ambiguous, such as those often encountered in the measurement of psychological features, and they can be used to assess behavioral changes, including therapeutic change [57,58,59]. Parallelly, the analysis of emotional variability requires studying the fluctuations in affective experience over brief timescales, also considering the speed and extent of these changes [60]. In this regard, the analysis of these fluctuations both in contexts and ecologically relevant and valid methods through the momentary ecological assessment (EMA) has proved to be particularly functional, because it allows us to ascertain and observe these dynamics as they take place [61]. Indeed, ecological monetary assessment can be extremely useful in assessing the temporal fluctuations in the variables of interest, thanks to its short and repeated observations [62]. The theoretical background of this study relies on specifically dynamic approaches to self-awareness and self-evaluation [63].

Based on this, the present article aimed at presenting a pair of quantitative case studies and applying both classical and nonlinear data analytic techniques to EMA data to summarize and then examine the temporal dynamics of both traits and affect over brief periods of time. It extends previous methods for studying personality dynamics to provide an illustration of the potential utility of nonlinear data analysis techniques to describe within-person variability.

## 2. Materials and Methods

### 2.1. Participants

Data are reported here for two American participants fluent in English. They were social science students and were recruited from an introductory psychology undergraduate participant pool and offered experimental credit, as well as additional financial compensation due to the relatively long duration (up to one week) of the study. Participant 1 (anonymously named Bob) is a 19-year-old white male, and participant 2 (Alice) a 20-year-old white female.

### 2.2. Measures

Measures were used in their English version and were administered in paper and pencil format at the beginning and end of the study, and also using EMA techniques.

#### 2.2.1. Ten-Item Personality Inventory (TIPI)

The ten-Item personality inventory (TIPI) is a ten-item measure of the Big Five (or five-factor model) dimensions [64]. The items are presented with a stem of “I see myself as…” and paired descriptors (e.g., “Extraverted) rated on a seven-point Likert scale, from 1 (disagree strongly) to 7 (agree strongly). Gosling and colleagues [64] reported good convergence between the scales and the Big Five inventory and the revised NEO personality inventory. Test–retest reliability of the TIPI after 6 weeks testifies to the stability of the measure (ranging from Extraversion = 0.77 to Openness to Experience = 0.62). In the current study the TIPI was given in paper and pencil form at the beginning and end of the study, as well as being administered regularly by the Palm Pilot Personal Digital Assistant (PDA).

In the PDA format participants used a “slider widget” with no numerical anchor points to respond to a prompt and a stem that said, “Right now I would describe myself as….” The bottom of the scale was labeled with “Disagree” and the top with “Agree,” different from the “Disagree Strongly” to “Agree strongly” paper and pencil anchors. This method solicited instantaneous trait ratings not tied to trait-related behaviors by the instructions, as reported by Fleeson and Gallagher [12]. For the TIPI items, the position of the widget from bottom to top was converted to a number (from 1 to 100) by the PDA EMA software. To maintain comparability in the variability of each scale, the original PDA 1 through 100 units was used for all scales (including the paper and pencil versions) in the tables and figures presented here.

#### 2.2.2. Positive and Negative Affect Schedule (PANAS)

The Positive and Negative Affect Schedule [65] consists of 20 affect terms to which participants respond on a 5-point Likert-type scale. On the paper and pencil version, participants were instructed to rate their affect “*during the past few weeks*.” The PANAS Pleasant (or Positive) affect scale is computed as the sum of ratings (prorated for missing items) on nine adjectives such as “Strong” and “Enthusiastic”; the Unpleasant (or Negative) affect scale is computed as the sum of 10 adjectives including “Distressed” and “Nervous”; the adjective “Interested” was omitted from the Positive scale per the developers of the instrument. The PDAs 1 through 100 units are used in this paper.

The PANAS has shown good psychometric properties and respectable test–retest correlations [65] (p. 1066). Stability coefficients depend on the time-frame for the PANAS rating, from “Moment” to “Year” or “General,” and these have ranged from a low of 0.39 for Negative Affect today to highs of 0.68 and 0.71 for Positive and Negative Affect “in general.” Notably the test–retest coefficients over 8 weeks for “moment” ratings (which most closely correspond to the PDA ratings used in this study) were 0.54 and 0.55, low but not unusually so and suggestive of some degree of temporal stability. The 20-item PANAS (with “during the past few weeks” instructions) was given in paper and pencil format at the beginning and end of study as well as being administered regularly in PDA form (with “right now” instructions). The screen provided a prompt of “Right now I am feeling….” and then the PANAS adjective. The bottom of the slider widget scale was again labeled with “Disagree” and the top with “Agree,” somewhat different from the “Very slightly or not at all” to “Extremely” anchors of the paper and pencil version.

### 2.3. Procedures

The Ecological Momentary Assessment portion of this study was conducted using Palm Pilot Personal Digital Assistants (PDAs) using the free iESP software [66], which was adapted from ESP, an earlier program. The original ESP software was developed by Dr. Lisa Feldman Barrett and programmed by Daniel J. Barrett [67]. This software was freely available, although it is no longer maintained and has now been supplanted in the rapid rise of cell-phone-based assessment software (e.g., [68]).

Bob and Alice were each given a Palm Pilot, charger, and instructions at the beginning of the study after filling out demographic information and paper and pencil versions of the TIPI, PANAS. The PDA was active for a 12 h period each day, a period chosen by the participant (different on weekends). Prompts occurred randomly over the active periods, approximately twice an hour. Prompts could be both visual and auditory. Bob and Alice were able to skip prompts and silence the PDA, for example when they were in class or otherwise occupied. The data collection lasted approximately one week (with some interruptions for software problems).

**Figure 1:**
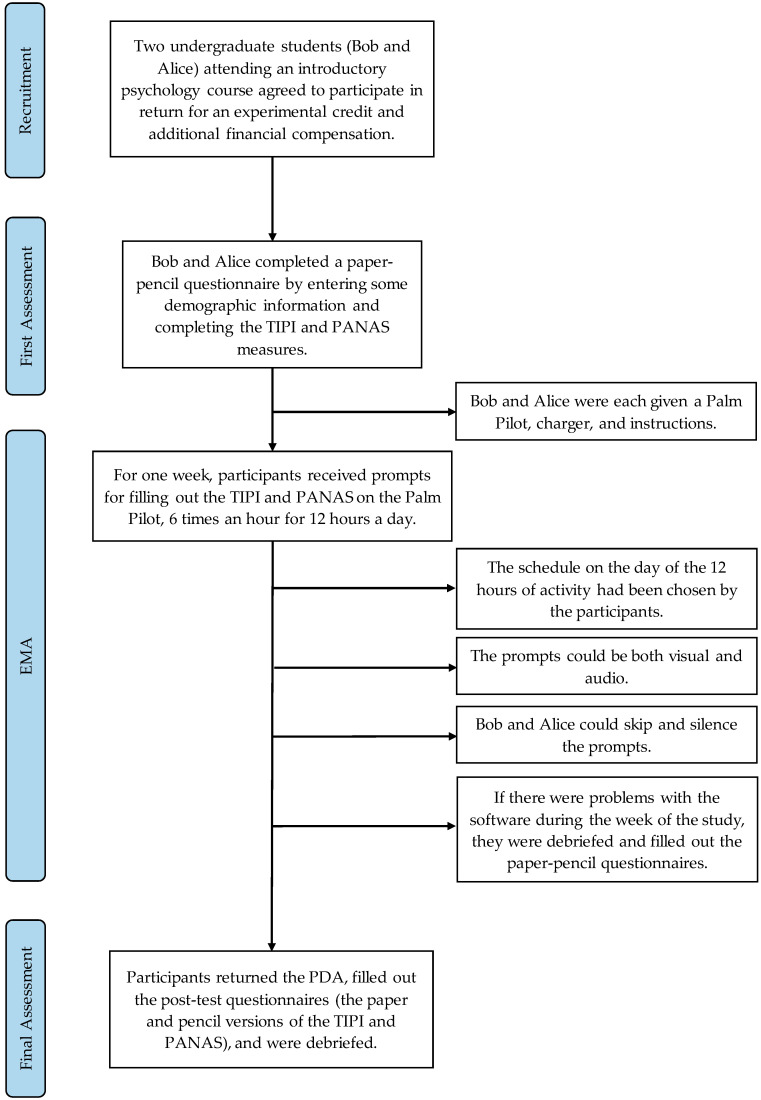
Flowchart illustrating the research technique.

At each prompt, participants responded to 30 items, the 10 items from the TIPI and 20 from the PANAS. Their responses, as well as date and time information, were recorded on the PDA; in the current study no information was gathered about the context or situation surrounding the response. At the end of the study (and sometimes at points during the week of the study if there were problems with the software) participants returned the PDA, filled out the paper and pencil questionnaires, and were debriefed. The data were then downloaded for analysis by “syncing” the PDAs. The number of useable data points for each participant was 74–76 (depending on the scale) for Bob, and 102 for Alice. At the end of the EMA period, participants returned to the lab, filled out paper and pencil post-test instruments, and were thanked and provided with the incentives for their participation (see Figure 1).

### 2.4. Data Analyses and Presentation

Since we were interested in comparing the variability of different scales, the PANAS scores (potentially ranging from 1 to 5) and the TIPI (potentially ranging from 1 to 7) were converted for comparison purposes and to have the same scale for the two measures: The original 1 to 100 scores from the PDA software were used in order to allow visual comparison and comparable summary statistics of central tendency and variability (scores of 1 on the TIPI and 1 on the PANAS correspond to scores of 1 on the 100-point PDA scale. scores of 7 on the TIPI and 5 on the PANAS correspond to 100 on the PDA scale; scores of 4 on the TIPI and 3 on the PANAS, the midpoints of the scales, both correspond to 50.5 on the PDA scale). The paper and pencil TIPI and PANAS scales are also presented in this 1-to-100 format.

Time series plots were produced for the 10 TIPI items and the two PANAS scales, Positive and Negative Affect. In addition, the standard deviation was computed as an index of variability and is reported along with minimum, maximum, and mean. In order to characterize the possible patterning in the data and to demonstrate the application of nonlinear data analyses techniques, phase plots are presented for sample scales and attractor reconstruction [58] was conducted using the *Chaos Data Analyzer* (*Professional Edition* software; American Institute of Physics, New York, NY, USA).

## 3. Results

### 3.1. Classical Variability Indices

**Figure 2:**
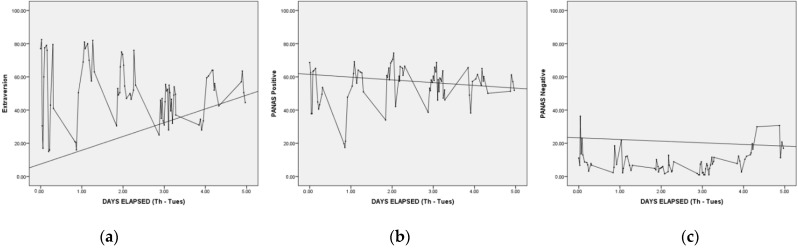
(**a**–**c**). *Variability in Extraversion and PANAS Positive Affect, Bob.* Notes: Straight line connects pre and post paper and pencil measurements. All measures expressed on 1-to-100 scales.

**Figure 3:**
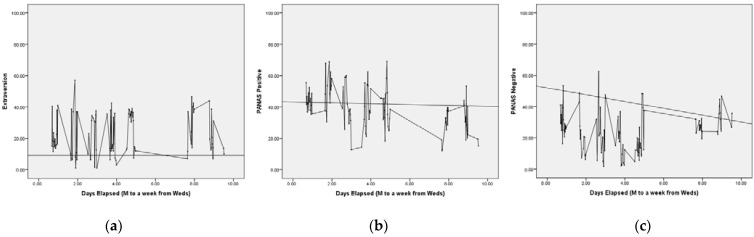
(**a**–**c**). *Variability in Extraversion and Positive PANAS Affect, Alice.* Notes: Straight line connects pre and post paper and pencil measurements. All PANAS and TIPI scales expressed on 1-to-100 scales.

Time series plots for each participant’s ratings of Extraversion on the TIPI and PANAS Negative affect scales are presented in Figure 2a,b and Figure 3a,b. Summary statistics are presented for all scales, presented in Table 1 and Table 2 (time series and phase portrait plots for all scales and for both participants are available from the first author).

Degree of variability was assessed both visually and quantitatively. On the basis of visual inspection, Bob and Alice showed striking fluctuations across time in both their affect and trait ratings. For Bob, ratings of affect were somewhat less variable (for PANAS, Mean *SD* = 9.3; Mean Range = 46.1) than his ratings of personality (for TIPI, Mean *SD* = 12.8; Mean Range = 57.5). Alice’s ratings of affect were also somewhat less variable (PANAS, Mean *SD* = 13.2; Mean Range = 58.8) than her TIPI personality ratings (TIPI, Mean *SD* = 14.4; Mean Range = 64.4). Overall, Alice showed comparatively more variability than Bob.

In order to have a different idea of the magnitude of these variations, participants’ ratings were converted to *z* scores based on their own means and standard deviations. Bob’s ratings fell in a range between −3.5 and +3.9. For Alice, the variation assessed in this way was similar, with all scores between −0.8 and +3.0. In general, the means of the PDA scores fell close to the paper and pencil values at the beginning and end of the study.

Based on both visual inspection and examination of SD as a variability indicator [12], it is striking that the degree of variability of the trait indicators, contrary to what would be expected from traditional trait theory (and the known fluctuations of emotions), is approximately as substantial as that of the PANAS scales. Despite their general reputation as trait and as state properties, respectively, the personality and affect ratings show very similar variability across time, consistent with the work of Fleeson and Gallagher [12].

### 3.2. Attractor Reconstruction and Evidence for Patterning and Possible Chaos

**Figure 4:**
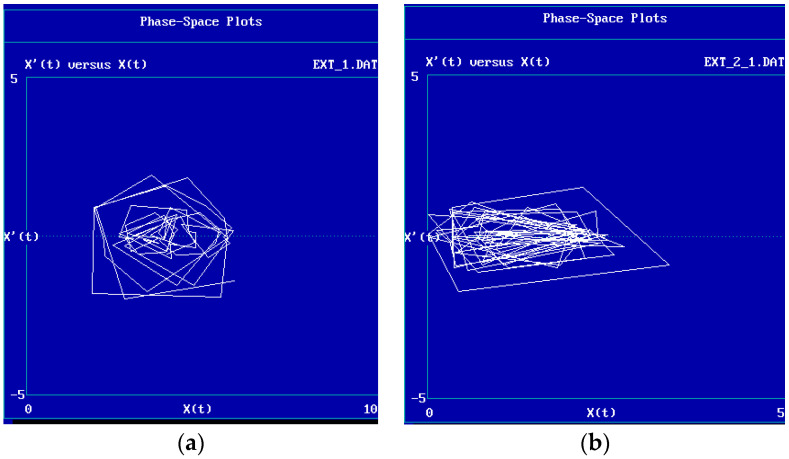
(**a**) *Example of attractor reconstruction: Extraversion ratings a. Bob (Data correspond to* Figure 2*a).* Notes: *n* = 76 valid points; estimated largest Lyapunov exponent: 0.585 ± 0.183; estimated correlation dimension: not computable. (**b**) *Example of attractor reconstruction: Extraversion ratings a. Alice (data correspond to* Figure 3*a).* Notes: *n* = 102 valid points; estimated largest Lyapunov exponent: 0.82 ± 0.16; estimated correlation dimension: 3.78 ± 1.25.

An attractor refers to a region in the space of a system’s possible conditions where the system tends to go and tends to linger. In a strange attractor, a sign of chaos in behavior, the system’s behavior is bounded yet ever novel, never both being in the same place and going the same direction twice. As a demonstration of promising techniques for looking at possible attractors in the dynamic data produced by the participants, a demonstration of “attractor reconstruction” [69] was conducted with the Extraversion and Positive and Negative affect ratings from Bob and Alice using the Chaos Data Analyzer Professional Version software [70]. Note that the data points are assumed (wrongly in this case) by the Chaos Data Analyzer to be equally spaced. Figure 4a,b depict phase plots of these data, with trait rating on the *x*-axis and velocity (rate of change to the next point) on the *y*-axis. This allows us to visualize the changing states of the person, as trait ratings substantially fluctuated yet remained bounded.

The shapes of these phase space plots are consistent with attractors and with the presence of low dimensional chaos in these data. In addition, the values of two parameters computed by the software, the largest Lyapunov exponent and the correlation dimension (an index of “degree” of chaos), are also compatible with chaotic fluctuations in this participant’s ratings of mood and five-factor model personality characteristics. However, as Sprott [71] reminds us, reports such as this one are “littered with false claims of chaos”, and this information is only as illustrative. It should also be noted that in Alice’s case, an estimate of the correlation dimension could not be computed. A more nuanced approach to setting parameters at the beginning of the computation of such an index is recommended by some investigators; this paper used the software’s pre-set parameters to do a “batch” computation across the subjects, referred to elsewhere as a “meat grinder” approach to this sort of data analysis [59]; more subtle approaches are recommended. Issues of stability, interpretability, computation and computability, parameter settings, sources of error, and length of the time series for such dynamic statistics and the Lyapunov exponent, in particular, are not addressed in the illustrations presented here. There is divergent opinion over the number of data points needed for state space analysis, and the numbers of points used here are low.

## 4. Discussion

The present study aimed to propose two quantitative case studies for exploring the temporal dynamics of both traits and affect by using both classical and nonlinear data analytic techniques with ecological momentary assessment (EMA) data.

Concerning affect, the EMA method allowed for observing relevant variability in the measurements over the week. This supports the efficacy and usefulness of EMA in monitoring fluctuations in momentary affect during the daily life of subjects [63], appears in line with previous evidence, both in clinical [72] and non-clinical samples [48], and may have important practical implications in the development of tailored interventions. Indeed, recent studies highlighted how emotions may serve as a source of information about one’s life satisfaction [73], and, although in healthy adolescents it may be associated with higher levels of creativity [22], in clinical subjects, affect fluctuation has been associated with stress, sleep problems [74], and higher levels of mental illness [75]. These preliminary results also explore and provide data in favor of assumptions concerning within-person variability in trait ratings, contrasted with a strict behavioral disposition or trait view of personality, according to which dispositions remain invariant across situations. This is in line with the recent evidence of Wilson and colleagues [76], who use the EMA to explore variability in both affects and traits, also analyzing their association and noting that fluctuations in personality states cannot be reduced to simple consequences of fluctuations in state affects. Furthermore, these findings are also consistent with Fleeson’s research, indicating how individuals can manifest different behavioral content related to traits in fluctuating levels of trait-related behavior across different moments [12,16,20]. In principle, such variability of traits can be interpreted in the light of the processes that underlie stable individual differences in such behavior [15]; other authors also suggested the within-person variability is itself a normal and important personal characteristic, perhaps also related to positive characteristics [49,77]. This variability itself may be recognizably patterned, another meaning of consistency in personality and the way that we are recognizable to ourselves and to others across time. There are also mathematical tools for understanding this patterning; at this point, the study of personality turns to the study of dynamics.

Once fluctuation is accepted, the next questions concern the possible meanings of this fluctuation, beyond noise in one’s data. The phase space point of view, in which one looks for regularities (in the form of attractors) in this variability, points at the “mediating processes” of Mischel and Shoda [15]. We can start to understand these processes by examining the time dependence and patterning of behavior. From the point of view of data analysis, detection and reconstruction of attractors represent techniques different from a more traditional and static “behavioral dispositions” approaches for describing personality.

A “strange attractor” is a region in phase space representing the bounded but unpredictable behavior of a system in chaos; while generally staying within a bounded range, the chaotic system is never both in the same place and going in the same direction twice. This is related to the notion of fractals, generally beautiful patterns that represent a sort of cross section of a strange attractor, in which patterns repeat themselves at different scales and levels of magnification [78]. Fractal patterns are self-similar and recognizable at many time scales. In fact, individuals’ personality styles are detectable by observers across time intervals from the near microscopic to over a lifetime, evidencing consistencies in pattern both across and within periods of fluctuation. As McArthur [79] asked in a 1989 Society for Personality Assessment meeting, “Are we fractals”?

Examining the patterning of personality and how temporal dynamics underlie both stability and change is, thus, related to defining a person’s recognizable style. This points toward reconceptualizing what we mean by consistency, stability, boundedness, and regulation, and will allow us to bridge traits and dynamic processes [18].

### 4.1. Pitfalls

With regard to data analysis, the uncertain status of attractor reconstruction techniques was mentioned earlier. Visual inspection of time series plots can produce inaccurate assessment of variability and inspection of phase space plots to erroneous claims of chaos. For the computation of parameters indexing complexity in data (as well as measuring information and disorder), large numbers of measurements are generally assumed; the data acquisition procedures used here (EMA) resulted in a relatively small number of data points, which in the past, would have been considered too few for confident application of these nonlinear data analysis techniques. A number of workers are now putting forward techniques for analyzing small data sets (e.g., [80]). In addition, the uneven time intervals that are inherent in most EMA methods (unless data are gathered regularly over 24 h) cause difficulty for many data analysis techniques; a number of approaches are now developing for how to deal with missing data and interpolation when modeling dynamic systems [81,82].

### 4.2. Limitations and Future Directions

This study has several limitations that need to be kept in mind when interpreting the results. First, the involvement of only two subjects implies the need to be cautious in generalizing the results. Although this study offers useful preliminary outcomes and conceptual input, these results should be confirmed in future research through the application of the EMA method in larger samples. Moreover, participants’ age may have contributed to the obtained results. Future research might involve older participants or may recruit a sample with age diversity to explore this aspect. Furthermore, our results may have been influenced by variables not included in the assessment of this study, such as self-awareness [63] and positive emotional granularity [83]. Future research could include these and other factors as covariates to overcome this issue. In addition, these analyses were conducted on data kept in the archive and collected in the year in which the ethical approval was provided. Although instruments and methods that can still be considered innovative and current have been used, this aspect must be considered for a correct reading of the results. Finally, the use of short self-report measures is another limitation that should be highlighted. On the one hand, these questionnaires have advantages, because they allow the researchers a quick method in preliminary studies for finding questions to deepen in future research. Furthermore, previous evidence has shown the possibility of measuring some relevant constructs, even with a single item (e.g., [84]). On the other hand, the use of self-report tools could expose well-known biases (e.g., social desirability) and might not be fully effective to identify variations in self-descriptions of personality. Therefore, an important challenge for future research could be the use of more in-depth questionnaires integrated with other instruments (e.g., detection of physiological parameters, interviews, etc.) in a multi-method perspective.

## 5. Conclusions

### 5.1. The Opportunity to Consider Temporal Fluctuation as an Informative Variable

The traditional approach to variability in data has been to view it as “error” [54]; a criticism that can be made of the current approach is that the very information we are studying represents meaningless temporal noise or instability rather than interesting and meaningful personal vicissitudes. Current approaches that treat time as an important variable in psychological research not only suggest taking temporal fluctuation as representing information rather than error [77,85], but suggest viewing regularities and irregularities in temporal patterning and the “shape” of dynamics; phase space plots represent a helpful way to study within-subjects data in this way.

Until recently, research that studies variability tended to rely on classical summary statistics, such as the standard deviation, to capture variability. Time series approaches that examine correlations and trends, as well as frequencies, represented a step forward. Newer techniques of nonlinear dynamical system analysis allow progress to be made in investigating whether, in some cases, human behavior may be chaotic; characteristics of chaotic systems have been suggested as applicable to both everyday and extraordinary, healthy and unhealthy human functioning [31,59,86].

### 5.2. Temporal Fluctuations and Psychological Variables

Even psychological phenomena that we have historically considered as stable “trait-like” have the potential to exhibit fluctuations that are interesting and perhaps complex or chaotic. This is likely a consequence of the fact that behavior derives from linked nonlinear systems [58,59], implying that well-being must rely on changeable, self-regulative heuristics and ongoing “satisficing” [87], rather than algorithms guaranteeing solution. These heuristics are self-regulative but not classically homeostatic, and their life-enhancing irregularity, unpredictability, and boundedness may best be considered in general terms of mathematical stationary states [57], rather as balance, equilibrium, stability, or homeostasis, expanding the notion of what behavioral consistency means [18]. Elsewhere, we refer to such models as “Somewhat-complicated” because they do not require intricate construction; complex behavior can emerge from systems that are quite simple [59]. Future research in personality and other aspects of psychology will benefit from focusing on the modeling of such systems [57] and fitting empirical data to them.

## Figures and Tables

**Table 1:** Personality and affect variability: Bob (*n* = 74–76 observations).

Scale	EMA Values					Paper and Pencil	
	Min	Max	Mean	Range	SD	Pre	Post
**Extraversion**	**15.0**	**82.5**	**51.1**	67.5	**18.0**	**9.3**	**9.3**
Agreeableness	43.0	100.0	76.7	57.0	9.6	91.8	83.5
Conscientiousness	39.0	90.5	70.3	51.5	11.0	58.8	50.5
Emotional Stability	34.5	100.0	82.0	65.5	14.3	25.8	42.3
Openness	30.0	76.0	58.5	46.0	11.1	91.8	75.3
**PANAS Positive**	**17.4**	**74.3**	**5.4**	**56.9**	**11.5**	**50.5**	**31.2**
**PANAS Negative**	**1.0**	**36.3**	**8.9**	**35.3**	**7.1**	**43.1**	**40.6**

Note: Bold indicate significant values. All scales converted to 1-to-100 scales for this table. Time series plots for the scales in bold type are shown in Figure 2.

**Table 2:** Personality and affect variability: Alice (*n* = 102 observations).

Scale	EMA Values					Paper and Pencil	
	Min	Max	Mean	Range	SD	Pre	Post
**Extraversion**	**1.0**	**57.0**	**22.8**	56.0	**13.4**	**17.5**	**58.8**
Agreeableness	53.5	100.0	85.4	46.5	12.6	50.5	50.5
Conscientiousness	11.0	87.5	57.1	76.5	15.0	91.8	83.5
Emotional Stability	16.5	93.0	63.0	76.5	19.6	50.5	58.8
Openness	28.0	94.5	72.2	66.5	11.6	67.0	75.3
**PANAS Positive**	**12.1**	**69.1**	**39.5**	**57.0**	**13.2**	**61.5**	**53.2**
**PANAS Negative**	**1.7**	**62.3**	**23.6**	**60.6**	**13.1**	**23.3**	**18.3**

Note: Bold indicate significant values. All scales expressed on 1-to-100 scales for this table. Time series plots for the scales in bold type are shown in Figure 3.

## Data Availability

The data presented in this study are available on request from the corresponding author. The data are not publicly available due to privacy reasons.
